# Discounting in economic evaluation of healthcare interventions: what about the risk term?

**DOI:** 10.1007/s10198-020-01257-x

**Published:** 2021-02-22

**Authors:** Lars Hultkrantz

**Affiliations:** School of Business, Örebro University, SE-701 82 Örebro, Sweden

**Keywords:** Cost–benefit analysis, Social discount rate, Project risk, H43, I10

## Abstract

Results from economic evaluations of long-term outcomes are strongly dependent on the chosen discount rate. A recent review of national guidelines for evaluation of healthcare interventions finds that “the level of currently used discount rates seems relatively high in many countries”. However, this conclusion comes from a comparison to rates derived or observed for investments in safe assets, while rate of return requirements are typically considerably higher when investment involves risk. This paper reviews recent literature on how to account for project-specific risk in determination of the social rate of discount and discusses implications for economic evaluation of healthcare interventions. It concludes that the available empirical evidence strongly suggests that the demand for and consumer value of health and healthcare is co-variant with income, which therefore implies that there is a non-diversifiable risk component of health-related investment.

## Introduction

The long-term investment nature of many health-care interventions, such as vaccination programs or prenatal treatments, makes outcomes of economic evaluations strongly dependent on the choice of the discount rate. Both the finance literature and some recent research on social discounting put emphasis on how to adjust discount rates for project-specific risk but this aspect has hitherto been largely ignored within health economics. This paper gives a brief introduction to some new results within discounting theory and sets out implications for health-care analysis.

A recent overview of national guidelines on discounting in health economic evaluations in 24 countries by Attema, Brouwer and Claxton [1] shows that recommended rates vary between 0 and 5%.^1^ The most common rate is 5%, followed by 3%. Based on a review of theoretical literature,^2^ the authors argue that these rates are on the high side and that lower rates may be considered to be more appropriate. However, as shown in their overview, most national guidelines are based on either a “descriptive” opportunity cost or a “prescriptive” social time preference approach, which in the former case is based on “risk-free” market rates of long-term government bonds and in the latter case on a parametrization of the so-called Ramsey equation (that will be described below). In both cases, this means that the discount rate does not include a project-specific risk term accounting for the non-diversifiable risk of an intervention or investment.

A common justification for ignorance of the risk-term^3^ within public and health economic evaluations has for long been a famous claim by Arrow and Lind [5] that “the government undertakes a wide range of public investments and it appears reasonable to assume that their returns are independent”, thus implying that such risk can be diversified away. However, as several authors have recently emphasized, this claim is fallacious (e.g., [3, 6–9]) since these returns can be expected to be correlated with the overall macroeconomy, which means that some risk component is not idiosyncratic.^4^ Based on this observation, recent research has examined ways for determining risk-adjusted social discount rates, employing the consumption-based capital asset pricing model (CCAPM) [10–12], which is a standard “workhorse” model for such analysis. The purpose of this article is to give a brief introduction to some of this current work and to discuss how it can be applied to health economics.

The next section gives a background on the social rate of discount and Sect. 3 reviews two recently suggested approaches to risk differentiation of this rate. In Sect. 4 implications for discounting in healthcare is discussed based on some empirical evidence. A few conclusions follow in the final section.

## The social rate of discount

A planner that wants to get an appropriate discount rate for evaluation of a public-funded program has a basic choice between using a market-based rate or an estimate of the Social Rate of Time Preference (STP). The main argument for the first approach is that it will reflect the actual opportunity cost of the resources used in the public program, and the main argument for the second approach is that social preferences may not coincide with market revealed preferences.

The main difficulty in the application of the *market-based approach* is the multitude of rates to choose among. Rates come nominal or real valued, before tax or after tax, with a term structure, and vary depending on the risk of the underlying security in ways that still are not very well understood. In small open economies that are part of the global financial market the opportunity cost of public spending is predominantly the (pre-tax) global market rate. Jordà et al. [13] have recently compiled data on long-run returns on government bonds, short-term bills, equities and housing in advanced economies from 1870. They find that real safe returns in peacetime periods have been low, in the 1–3% range, but quite volatile, while the ex-post risky rate on both equities and housing has been quite stable averaging between 6 and 8%. In comparison, Attema et al. [1] observe that several countries recommend discount rates for health economic evaluations based on the cost of government borrowing, for instance the 5-year real risk-free government bond rate in New Zealand. A notable exception is the United States, which was not included in the overview. The US federal guidelines recommend 7%, which “approximates the marginal pre-tax rate of return on an average investment in the private sector in recent years” [14]. In fact, this seems to be the only country taking the market-based approach that includes a risk term representing (average) project risk.^5^,^6^

The *STP approach* has been developed along two lines in the theoretical literature: (1) the Ramsey model, which is based on deterministic conditions, and (2) the CCAPM model, which explicitly takes risk into account. The first has its origins in a study by Ramsey [15] that asked how much a nation should save. To answer, he set up a model where social welfare is expressed as the sum of a infinite stream of instantaneous utility $u\left( {c_{{\text{t}}} } \right)$ from consumption $c_{{\text{t}}}$ discounted at a constant rate $\delta .$ Maximizing the social welfare specified in this way results in a first-order condition that with some simplifying technical assumtions (iso-elastic utility and a constant rate of growth of consumption per capita) gives the Ramsey equation

1 $$r{ } = \delta { } + \gamma g,$$

where $\gamma$ is the elasticity of marginal utility with respect to consumption and *g* is the rate of growth of consumption per capita. The first term is the pure time preference, due to unequal weighting of the utility of different generations or impatience within a generation, or both. The second term takes care of the fact that a long-term investment in a growing economy is a transfer from the poor (current generation) to the rich (subsequent generations), and adjusts the discount rate in proportion to the expected relative decrease of the marginal utility of consumption due to growth in consumption per capita.

The second model, the CCAPM, is derived in a framework where a representative consumer maximizes the total expected utility from current consumption $u\left( {c_{0} } \right)$ and expected future consumption ${\text{Eu}}\left( {c_{{\text{t}}} } \right)$ discounted with the utility discount rate $\delta .$ Again applying some simplifying technical assumptions (iso-elastic utility, growth is a Brownian motion and returns and the log of per capita consumption are jointly normally distributed) to the first-order condition gives the CCAPM equation:

2 $$r_{i} = r^{f} + \beta_{i} \pi ,$$

where the first term on the right-hand side is the risk-free rate of discount and the second term is the risk term for a specific project *i*. This term is the product of the project beta and the average risk premium $\pi$. The project beta is a measure of how the total consumption per capita risk (volatility) is affected by the project. It is defined as the covariance between the project returns and overall consumption per capita over the variance of consumption per capita. It can be noticed that total consumption can be regarded as the returns from all individual projects (including investment in human and natural resource capital), which implies that the average beta is unity. As a large part of total consumption in modern economies is public consumption this implies that it is unlikely that project beta of an average public investment is zero, in contrast to the previously cited claim made by Arrow and Lind [5].^7^

An obvious difference between the Ramsey and CCAPM models,^8^ is that only CCAPM accounts for the fundamental fact that the future is not just happening at another time than the present but also uncertain.^9^ In the standard simple «Gaussian» representation given above uncertainty is measured by the standard deviation, which means that other moments of the probability distribution, possibly giving rise to skewness and fat tails, are disregarded. This is important because it can be shown that this implies that the risk premium $\pi$ is proportional to the variance of consumption per capita, which with historical data and plausible values of the elasticity of marginal utility implies a level of the risk premium that is of an order of 0.1 relative to actual risk premiums on asset markets (which is «the equity premium puzzle», [17]). Several explanations have been suggested, for instance that stock investors have a strong aversion against risk in poor macroeconomic states, that is, «individuals fears stocks primarily because they do bad in recessions» [18], or that, as shown by Barro and Jin [19] the puzzle partly vanishes when fat-tail properties of the probability distribution (i.e., low-probability disasters such as stock-market crashes) are considered. However, recent evidence showing high risk-premia not just for stocks but also for housing seem to magnify the puzzle and reduce the scope for these explanations [20].

As noticed in the introduction, the overview of current practices in health economics by Attema et al. [1] suggests that guidelines in most countries focus on the risk-free component of the social discount rate, building on either risk-free government bond rates or parametrization of the Ramsey rule. In the remainder of this article, I will, therefore, discuss what insights current research building on the CCAPM framework can provide for discounting in health economics.

## Two approaches to risk-adjustment based on CCAPM

Research on risk-adjustment of social-discount rates is quite novel and many aspects remain to be considered. Here I will summarize two recent contributions that give some guidance into how to derive the risk term in specific cases: The tail-hedge gamma and the elasticity-based beta. The centre of analysis is the correlation between returns from a portfolio of investment objects or specific programs within a sector and the overall macroeconomy.

### The tail-hedge gamma

Weitzman [21, 22] has developed a model for calculation of the social rate of discount for investments in risky assets. He argues that in consideration of real-world risk, risk-averse investors expect that low-probability large loss events are somewhat more likely than what is implied by the normal distribution. He shows that with otherwise standard assumptions on the parameters of the Ramsey equation it is possible to reconcile the Ramsey equation with empirically observed levels of the risk-free rate and equity premium.

He then assumes that the undiscounted net benefit of an investment can be decomposed as a linear combination of contemporary consumption and a project-specific random variable that is uncorrelated with consumption (which therefore can be made deterministic by diversification over a pool of projects). He shows that the discount rate for a project will be an average of the risk-free rate and the rate of a risky investment, where weights are given by the discount factors and “the *fraction* of expected payoffs that on average is due to the uncertain macro-economy” ([21], p. 15, italics in original).

Weitzman does not indicate how this decomposition can be empirically estimated. This issue has recently been explored in [23–25]. An especially useful result from [24] is that if projects returns and aggregate consumption follow Brownian motion and are co-integrated then the fraction of the risk rate is unity, i.e., the discount rate equals the risk rate. Consistent with this, it is well established in empirical research that long-term relationships between stock prices and GDP or dividends and GDP are co-integrated [26].^10^

This model is developed primarily for the purpose of analysing catastrophic risks and not investment risk associated with variation within “normal” boundaries. In addition, as shown by Weitzman, the assumptions of the model imply that short-term beta is one or below one, which clearly is very restrictive and could mislead investors in “normal-risk” cases. However, the take-home message from this analysis for the health sector is that there may be reasons to use a low discount rate in the evaluation of long-term investments that provide protection in catastrophic events.

### Elasticity-based beta

A recent study by Cherbonnier and Gollier [28] addresses the task of estimating CCAPM beta for public investment in a novel way. They assume that investments are made that enable production and consumption of services and that demand and supply relations that can be described by log-linear equations in price and income, thus by constant elasticities. On the demand side, the consumers’ instantaneous benefit of the service is

3 $$B = \vartheta C^{\rho } \frac{{x^{1 - \alpha } }}{1 - \alpha },$$

which gives the following demand function

4 $$\ln x^{d} = \frac{1}{\alpha }\ln \vartheta + \frac{\rho }{\alpha }\ln C - \frac{1}{\alpha }\ln p ,$$

where $x^{d}$ is demand, *C* is income (or aggregate consumption), *p* is price, $\frac{\rho }{\alpha }$ is the income elasticity of demand, $\rho$ is the income elasticity of the consumer valuation (willingness to pay), and $- \frac{1}{\alpha }$ is the price elasticity ($\alpha < 1)$. A similar log-linear equation is specified for the supply, however, the main attention is paid to the three special cases: Case 1 supply is perfectly inelastic, Case 2 supply is perfectly elastic, and Case 3 supply is perfectly inelastic but with a limited capacity.

Assuming that growth of C is Brownian motion with a linear trend, and variable cost is Brownian motion, Cherbonnier and Gollier [28] show the following results for the CCAPM beta in the three special cases:

1. 1 (perfectly inelastic supply): constant beta, $\beta = \rho$
2. 2 (perfectly elastic supply): constant beta, $\beta = { }\frac{\rho }{\alpha }$
3. 3 (limited capacity): declining beta, from $\beta = \rho$ to $\beta = { }\frac{\rho }{\alpha }$

Notice that since $\alpha < 1$ the income elasticity of demand is by assumption higher than the income elasticity of the willingness to pay. Thus these results tell that in the combined case 3, which for instance could represent an infrastructure investment where services are supplied at a constant marginal cost up to the capacity limit, then beta for short maturities is close to the demand elasticity with respect to income and for long maturities approaches the willingness to pay elasticity with respect to income.

In interpreting these results it should first be observed that, in a growing economy, a high-income elasticity of benefits means, on the one hand, a more rapid growth of the nominal (undiscounted) benefits, on the other hand a higher beta, and therefore a higher risk-adjusted discount rate. The net effect on the present value can go in either direction [28, p. 7].^11^ The polar cases 1 and 2 have different implications for how income growth affects the quantity and price of the service. In case 2, with perfectly elastic supply, price will stay constant but the quantity demanded will vary proportionally to income, at a degree equal to the income elasticity of demand. In the polar case 1, quantity will be constant but price will vary, with the income elasticity of the willingness to pay as the factor of proportionality.

## Implications for discounting in healthcare

Obviously the covariance between health care consumption and for instance GDP per capita may vary a lot across different types of health care purposes. Cherbonnier and Gollier [28] remark that one implication of their analysis is that the risk term will have the same sign as the income elasticity, and they suggest that there may be examples of inferior goods, associated with a negative risk term, within health. One can think of the capacity for health care during disasters that strike both the economy and health. However, here we will focus on the level of total national expenditure on health, that is, implications for general-purpose investments that provide the means for the supply of a range of health-care services, such as education of physical practitioners.

A first question is then if there is a positive “tail-hedge gamma” that would make it possible to separate a risk-free component of returns on such investments. As noticed above, this can be answered by co-integration analysis. A quite common finding in the literature (e.g., [30, 31]) is that total healthcare expenditure is co-integrated with GDP per capita, thus indicating that separation of a linear risk-free component of returns is not possible at this aggregation level.

Next question concerns the size of the beta in a CCAPM. In the model of Cherbonnier and Gollier [28], it is at least as high as the income elasticity of demand. Another theoretical prediction [32] is that this parameter should exceed unity, the reason being that spending on health to extend life allows individuals to purchase additional periods of utility. This means that unlike the marginal utility of consumption of other goods, the marginal utility of life extension does not decline.^12^ This implies that health care is a luxury good, leading to increasing GDP share spending on health care. And indeed this is what generally can be observed in the statistics, for instance the average GDP share of the OECD35 countries rose from 8 to 9% from 2003 to 2016 [33, Ch. 7]. However, this may partly be due to the aging of the population, giving rise to higher health-care needs simultaneously with income growth. Chakroun [34] finds that it may so be and that controlling for this factor, the income elasticity is slightly below unity for most OECD countries. Another study with a similar finding is Acemoglou, Finkelstein and Notowidiglo [35] that uses an instrument-variable approach and reports a central-value estimate for this parameter at 0.7. However, they examine hospital expenditure and not total health expenditure which may lead to an underestimation.

Studies using cross-sectional data on individuals typically have found low or zero income elasticity, but that may be caused by omitted-variable bias, as people with high income may have fewer health problems. A recent study with such data that uses a natural experiment (the “social security notch” in the US) to overcome this problem is Tsai [36]. She finds that out-of-pocket medical expenditures of the elderly have income elasticity close to one, and significantly exceed unity among elderly with low education.

Hammit and Robinson [37] review some studies of the income elasticity of the value per statistical life (VSL), i.e., a measure of the willingness to pay for health. They find that, as for the income elasticity of demand, cross-sectional micro-level data typically reveal very low magnitudes, but longitudinal studies, cross-country comparisons and estimates from quantile regressions all suggest that the income elasticity of VSL is greater than unity. However, more recently Masterman and Viscusi [38] conclude from a meta-analysis of findings from stated-preference studies of VSL that the income elasticity is 0.55 for rich countries and 1.0 for low-income countries.

## Conclusion

There is a wide discrepancy between the literature on discounting in the finance and macro-finance literature and the current practices and guidelines for the economic evaluation of healthcare. This has recently been noticed also by Claxton et al. [39]. As shown by Attema et al. [1] health-economics guidelines consistently ignore the risk term of the social discount rate. It can be noticed that these guidelines have until recently been in full agreement with the treatment of this issue in several standard textbooks on cost–benefit and cost-utility analysis, for instance Brent ([4], Ch. 7). The fifth edition of Boardman et al. [2] though comments in passing that “Finally, some authors argue that the risk-free rate should be adjusted for the particular risk of a particular project” (p. 247). Possibly, an indication that the tide is changing is the recently updated edition of OECD’s guidelines on “Cost-Benefit Analysis and the Environment” [3] that now include a review of some literature on risk-adjustment of social discount rates.

In contrast, the upshot of the macroeconomics and finance literature is that the discount rate includes a risk term that is the product of a general risk premium and a factor (“beta”) that reflects investment-specific risk. While this is standard in the analysis of private investment is also applicable to the evaluation of public investment, such as public-funded health measures, and therefore to the social rate of discount.

Further, the available empirical evidence strongly suggests that the demand for and consumer value of health and healthcare is co-variant with income, which therefore implies that there is a non-diversifiable risk component of health-related investment. However, it is important to observe that in a growing economy this association affects both the numerator (the stream of undiscounted net benefits) and the denominator (the discount factor) of the net present value expression.^13^ Another way to put this is that if the undiscounted benefit of an investment is expected to grow exponentially at a constant rate *g* during the life of the investment, then the present value of benefits can be computed from the initial benefit *B* and the effective discount rate *r-g*.^14^ Both *r* and *g* will be affected by income growth.

One purpose of the review article by Attema et al. [1] was to propose “a research agenda with topics that deserve special attention in the search for improved discounting guidelines” (p. 755). In conclusion, of this comment to their review, I want to add to their list the need for research on how to estimate relevant magnitudes of investment-specific risk-terms of the discount rates.

## Footnotes

1 Boardman et al. [2] and Atkinson et al. [3] present compilations of recommended discount rates in national guidelines for benefit–cost analysis of public investments in a range of (mostly OECD) countries.

2 A comprehensive textbook treatment of the state of the art with focus on health-economics applications is in Brent ([4], Ch. 7).

3 Notice that the motive for a risk term is that uncertainty comes with a cost, i.e., people are risk averse and therefore are willing to pay more for a certain outcome than for a lottery with an equal expected (probability-weighted) outcome, and further that this difference increases with the magnitude of the uncertainty (or risk). Economic evaluations regularly report and analyse uncertainty of results by calculation of confidence intervals, sensitivity analysis, break-even analysis, and sometimes option-value analysis. The cost of uncertainty (or risk) can be included in the analysis in several ways, for instance by replacing uncertain outcomes with certainty equivalents (with a lower value), but here we focus on the standard procedure, which is to add a risk term to the discount rate.

4 This is in fact stated by Arrow and Lind [5], remarking that the results “depend on returns from a public investment being independent on other components of national income” (p. 373). Probably, the reason that this has gone unnoticed by many is the analogy often made to the more well-known risk pooling in insurance (that is, when a large collective of individuals share evenly the burden of some unfortunate outcome, for instance a house fire). But this popular analogy ignores that insurance typically covers costs of events that are uncorrelated with national income. Also, some may think that returns from public investment are mostly associated with collective consumption such as defense and that the value of this is not income elastic. However, a large part of government consumption is individual consumption (63% in 2019 in the EU), and also many benefits of collective consumption such as access to roads are correlated with national income (for instance, the value of travel-time savings).

5 This applies to”programs that provide benefits and costs to the general public”. For “cost-effectiveness analysis of internal planning decisions of the Federal Government” the guidelines instructs use of a risk-free real rate derived from rates on government bonds [14].

6 However, “risk-free” rates may be affected by overall macro-economic risk. For example in the Ramsey equation (se Eq. 1 below), the term representing “pure time preference” may be affected by consideration of the possibility of future global catastrophes (such as a hit by a comet). For example in the UK such catastrophe risk is explicitly taken into account.

7 Baumstark and Gollier [7] make this point.

8 There is a seemingly close correspondence between the Ramsey and the CCAPM models in an economy where the consumer is a representative agent that therefore also can be seen as the social planner. However, the parameter γ has different interpretations in these models. In the Ramsey model 1/*γ* is the elasticity of intertemporal substitution while in the CCAPM γ is the coefficient of relative risk aversion. These are separate aspects, though both relate to the curvature of utility functions.

9 Except for in the way that is mentioned in note 6.In this brief overview I have not mentioned “the augmented Ramsey rule”, which is an extension of Eq. (1) that accounts for the volatility of macroeconomic growth (the *g* parameter in Eq. (1)), see Gollier [16]. One reason is that I want to focus on project risk, another that this extension is of practical importance mostly for very long time horizons (such as in climate policy), see Gollier [16]. To give a numerical example of the magnitudes involved, consideration of project risk can affect whether to add, say, a 4% risk term to the risk-free rate. In contrast, the macro-economic risk component of the augmented Ramsey rule is in a calibration to United States annual data found to reduce the discount rate by 0.4% point [16, p. 48], thus suggesting that project risk is an order of magnitude more important.

10 This is consistent with standard macroeconomic theory, although an exception may be the case of a small open economy with fixed exchange rates [27].

11 As an example, Dietz et al. [29] in an analysis of the investment-specific risk of climate-change mitigation find that the net present value of carbon emissions abatement is increasing in the”climate beta”, as the increase of the undiscounted stream of benefits dominates the discounting effect.

12 However, as pointed out by an anonymous reviewer, when diminished health-related quality of life due to advanced age is taken into account, it is not credible that the marginal utility of life extension does not decline.

13 This connection is not made clear by Claxton et al. [39].

14 The present value of a growing annuity is $PV = \frac{B}{r - g}[1 - \left( {\frac{1 + g}{{1 + r}})^{N} } \right]$ where N is the life length of the investment.
